# The Role of Working Memory on Writing Processes

**DOI:** 10.3389/fpsyg.2021.738395

**Published:** 2021-08-27

**Authors:** Francesca De Vita, Susanna Schmidt, Carla Tinti, Anna Maria Re

**Affiliations:** Department of Psychology, University of Turin, Turin, Italy

**Keywords:** working memory, writing speed, orthographic skills, expressive writing, children, updating

## Abstract

Literature has extensively demonstrated the coordination role of working memory (WM) in complex tasks such as writing. However, previous studies mostly concentrated on the relation between passive WM (e.g., WM span) components and specific writing tasks (e.g., dictation). Here, we aimed to investigate the relationship between different writing skills and the performance on a WM updating task measuring the more active components of WM. From a pool of 160 Italian pupils (grades 3–5), we selected 46 children divided in two groups based on their WM updating performance. The first group consisted of 21 children with low WM updating performance (≤10th percentile), the second group consisted of 25 children with high WM updating performance (≥90th percentile). All children were tested on a battery of writing tasks to assess writing speed, orthographic skills, and competences in expressive writing. MANOVAs and a discriminant analysis were computed to assess group differences and the contribution of the different writing tests in correctly predicting group membership. The results revealed that children with high WM updating performance scored significantly higher than children with low WM updating performance on most of the writing tasks. These results highlight the relevant role of the active components of WM on writing processes. In addition, they suggest that the improvement of writing skills should rely not only on the training of the specific processes implied in this complex task, but also on the training of the cognitive processes that support them, such as active WM processes.

## Introduction

Working memory (WM) plays an important role in many cognitive tasks (e.g., Baddeley, 1983, 1986, 2000; Cornoldi, 2007, 2019), and several authors focused their attention on its fundamental coordination role in interactive and recursive learning processes such as writing (e.g., Kellogg, 1987; Bourdin and Fayol, 1994; Butterfield et al., 1996; McCutchen, 1996, 2000; Swanson and Berninger, 1996; Berninger, 1999; Cornoldi et al., 2010; Re et al., 2014; Capodieci et al., 2018; Cornoldi, 2019).

According to Baddeley (2000), WM is “a limited capacity system allowing the temporary storage and manipulation of information necessary for such complex cognitive tasks as comprehension, learning, and reasoning” (p. 418). More specifically, Baddeley’s model (2000) theorizes the existence of a “central executive” system, an attentional component that controls the flow of information to and from lower-order short-term “slave systems” with limited storage capacity (i.e., visuospatial sketchpad, phonological loop, and episodic buffer). Empirical findings (e.g., Alloway et al., 2006; Martinussen and Major, 2011) indeed suggest that there are different levels of performance in children when they perform tasks that mainly require attentional resources compared to tasks that merely rely on storing information in short-term memory systems, supporting Baddeley’s hierarchical component model of WM. On the other hand, Cornoldi and Vecchi (2004) suggest that the distinction between more passive and more active WM processes can vary along a continuum. More specifically, the authors represent the WM model as a cone in which the horizontal plane contains the types of information (e.g., visual or spatial), while the vertical plane represents executive/attentional control. The more a task or skill is automated, the lower the engagement of WM, the more a task requires non-automated processes, the greater the engagement of WM.

Based on these premises, the main purpose of this study is to investigate the relationship between the more active components of WM and different writing skills in Italian third – to fifth-graders.

Learning to write is a process that necessitates the development of several skills that are likely to involve different amounts of WM resources: graphomotor skills, which underlie the production of the graphic stroke; orthographic skills, which involve orthographic knowledge and the correct conversion of phonemes into graphemes; and written expression skills, the most complex, which are necessary to produce a written text (Ferrara and Cornoldi, 2019). The latter involve several steps: planning (goal definition, idea generation, organization of ideas, themes, and logical conclusions), transcription (converting the mental representation of the text into written symbols using phonological and orthographic knowledge), and revision (checking for errors and inconsistencies, making adjustments to improve the text; Hayes and Flower, 1980; Swanson and Berninger, 1996; Cornoldi et al., 2010).

The complexity of a task such as writing crucially involves WM (Berninger, 1999; Swanson and Siegel, 2001; Carretti et al., 2013b; Cornoldi, 2019), since WM allows to keep in memory the information necessary to generate and to transcript a sentence and to monitor the execution of the writing process (McCutchen, 1996; Swanson and Berninger, 1996; Cornoldi et al., 2010). In particular, WM seems to predict children’s (grades 4–6) performance in text creation better than performance in transcription, which relies more on automated processes (Swanson and Berninger, 1996).

Berninger (1999) points out the importance of adequate development of transcription capacity to reduce the burden of this process in terms of WM resources. Without adequate automaticity in the transcription process, most WM resources are devoted to graphomotor and orthographic aspects, resulting in a reduction of resources available for higher-order cognitive processes involved in text composition (Bourdin and Fayol, 1994; Cornoldi et al., 2010).

Other authors claim that sufficiently automated higher-order cognitive processes involved in the production of a written text, such as the processes of language encoding and a good general knowledge, would allow reducing the processing constraints imposed by the limited capacity of WM (e.g., McCutchen, 2000). Thus, if children develop a sufficiently automated ability in orthographic knowledge, knowledge about the topic of the text, the recipient, and planning, then they might be facilitated in reducing the burden on the limited resources of WM (Stanovich, 1990; McCutchen, 2000). In fact, according to McCutchen (2000), even though higher-order cognitive processes and knowledge stored in long-term memory contribute to writing ability, accessing and integrating multiple sources of information would not be possible without the coordination and control functions of WM and the WM works better if all the different processes are sufficiently automated.

As shown by Re et al. (2014) in a study involving a sample of fourth and fifth graders, WM plays a central role not only in spontaneous writing, but also in tasks such as dictation. In dictation, WM enables the maintenance of information and the recovery of the correct orthographic form of the dictated word and this is especially true in languages with opaque spelling or when writing irregular words.

In a language with transparent orthography, such as Italian, the phoneme-grapheme correspondence is respected and, in most cases, a correct phonological mental representation of a word is sufficient to avoid errors (Re et al., 2014), as it allows to correctly identify the individual phonemes and then transcribe them into the corresponding graphemes (Brandenburg et al., 2015; Ferrara and Cornoldi, 2019). But there are also irregularities in Italian, e.g., “cera” (wax), and “c’era” (there was): these two words have the same sound but are spelled differently, so they are homophonic but not homographic. These words, to be spelled correctly, require the use of the lexical-semantic route to access the orthographic and semantic representation of the word stored in long-term memory (Coltheart, 1981; Ferrara and Cornoldi, 2019). The lexical-semantic route is very commonly used in the transcription of familiar words because it speeds up the transcription process, although it places a greater burden on WM (Ferrara and Cornoldi, 2019).

The coordination ability of WM also influences writing speed in children (grades 4–5; Capodieci et al., 2018). Indeed, the execution of a handwriting task requires the integration of grapho-motor skills, transcription skills, and higher-order metacognitive processes (Hoskyn and Swanson, 2003; Peverly, 2006; Capodieci et al., 2018). The execution of specific grapho-motor movements, especially when they are not automated, and the simultaneous access to orthographic knowledge depends on the coordination processes of WM, and when the latter are not efficient enough, there are negative consequences also in terms of writing speed and readability of the produced text (Peverly, 2006; Capodieci et al., 2018).

To sum up, although in the presence of an adequate development of the multiple components that characterize writing ability, individual differences in WM can be hypothesized to explain differences in writing performance (Peverly, 2006).

The present study is rooted in work on the relationship between writing and WM. However, the studies reported in the literature mostly examined the relationship between written production and WM (e.g., McCutchen, 1996, 2000; Swanson and Berninger, 1996; Berninger, 1999; Cornoldi et al., 2010), or that between spelling skills and performance on tests of memory span (e.g., Bourdin and Fayol, 1994; Hoskyn and Swanson, 2003; Re et al., 2014; Brandenburg et al., 2015). Here, we aimed to examine the relationship between various writing tests (writing speed, spelling, and text production) and performance on a test of WM updating, since we were interested in the more active components of WM.

Working memory updating tests require remembering a certain number of items based on specific criteria that need to be continuously updated (Carretti et al., 2005, 2009). WM updating implies a constant modification of the information retained in memory, keeping only the items that are relevant and removing the irrelevant ones (Morris and Jones, 1990). Thus, the function of WM during an updating task is not the mere retention of information, but a dynamic manipulation of it (Morris and Jones, 1990; Carretti et al., 2005, 2009; Artuso and Palladino, 2016). Updating tests aim to measure this main function of WM, i.e., the ability to maintain and temporarily process information that is constantly updated (Morris and Jones, 1990; Carretti et al., 2005).

Given that most empirical studies that addressed the relationship between writing skills and WM used span-type tests, and that updating tests instead seem to reflect more the active role of WM, we wondered whether a significant difference in writing skills might be observed between children with high vs. low performance on an updating task. We hypothesized that children with lower performance on an updating task would also have greater difficulty on writing tests. More specifically, errors in updating tests seem to be due to a poor ability to coordinate attentional control resources (Carretti et al., 2009), so they should also be related to a poor ability to coordinate and adequately allocate WM resources across the different processes involved in writing. This should be particularly true for those writing tasks that require greater cognitive effort, such as written expression tasks or dictation, which need constant access to spelling knowledge. However, because writing speed tasks also require good functioning of WM for effective coordination between grapho-motor and transcription processes (Peverly, 2006; Capodieci et al., 2018), we hypothesize that children with low performance on updating tasks might also perform poorly on writing speed tasks.

## Method

### Participants

From an initial pool of 160 children (grades 3–5) attending a primary school in Turin (Piedmont, Italy), 9 children with attested cognitive disabilities and/or specific learning disorders were excluded and then 46 children were selected based on their performance in a WM updating task. These 46 children were divided into two groups. The first group consisted of 21 children (10 boys, mean age: 116.43 months, *SD* = 9.79) who showed a low performance in the updating task (≤10th percentile). The second group consisted of 25 children (20 boys, mean age: 121.32 months, *SD* = 8.34) who showed a high performance in the updating task (≥90th percentile). The two groups significantly differed in their performance in the updating task, *t*(44) = 21.35, *p* < 0.001, *d* = 1.22, while they did not differ significantly for age, *t*(44) = 1.83, *p* = 0.90.

### Instruments

Two instruments were used in the present research:

1. A standardized WM updating test taken from the CO-TT [Comprensione Orale. Test e Trattamento (Listening comprehension. Test and treatment), Carretti et al., 2013a; see Palladino et al., 2001; Carretti et al., 2013a, for more information on the reliability and validity of this test].
2. Writing tests taken from the BVSCO-2 [Batteria per la Valutazione della Scrittura e della Competenza Ortografica- 2 (Battery for the assessment of writing and spelling skills), Tressoldi et al., 2013] The BVSCO-2 is a standardized battery which has been tested for reliability and external validity (Cornoldi et al., 1999; Tressoldi and Cornoldi, 2000).

The CO -TT WM updating test consists of six lists of eight words each. The child’s task is to remember the three smallest objects in the list and write them down on a piece of paper.

For each list, a point is assigned for each correctly identified word reported in the correct order of presentation (max: 3 points for each list, range of the scale: 0–24). For instance, let us consider the following list: radiator, ambulance, piano, circus, bridge, washing machine, square, and castle. If the child writes “piano” and “washing machine” a score of 2 will be awarded, if s/he writes “radiator,” “washing machine,” “piano,” the score will always be 2 because only “radiator” and “washing machine” are remembered in the correct order (Carretti et al., 2013a).

The writing tests of the BVSCO-2 can be grouped in three sub-domains: tests of writing speed, tests of orthographic competences, and tests of expressive writing.

1. Tests of *writing speed* measuring the child’s grapho-motor skills in a given time. These are three tests, each lasting 1 min, and consisting in writing as many graphemes as possible in the allotted time. The first is writing “*le*” strictly in cursive, while the other two, writing “uno” (“one”) and writing “uno due tre.” (consecutive numbers in words), leave the child free to choose the character (block letters or cursive). The parameter considered is the number of graphemes correctly written by the child. These three tasks require the involvement of graphomotor skills and the last one also the access to orthographic knowledge.
2. Tests measuring the child’s *orthographic skills* in terms of percentages of spelling errors made in each of four tasks:

The first three tasks are *dictation of a text, of words and non-words*, in which the child is asked to write what is dictated by the examiner.
  In the fourth task, *dictation of sentences with homophonic non-homographic words*, the child is asked to write sentences such as “Sul pavimento non *c’era* la *cera*” (There was no wax on the floor).
The child is free to write with the character s/he prefers (block letters or cursive). These four tasks require the access to orthographic knowledge.

3. Two tests aimed at measuring skills of *expressive writing:* description and narration. The description test asks the child to describe a colored image (a single image which represents a specific situation), the narration test asks the child to write a story based on colored vignettes (a series of images which represent a brief story). For both tests, the child has 10 min at disposal. Performance is measured by the number of words written and the percentage of orthographic errors. These two tests require the involvement of higher cognitive processes: planning, transcription and revision.

### Procedure

After obtaining permission from the school principal and the children’s parents to participate in the research, a meeting was held with the teachers to present them with the research objectives and to plan the testing phase. Then trained research assistants went to each class to run the entire battery of tests collectively to all students in a single session that lasted about two and a half hours including 3 breaks of about 15 min. The WM updating test was always administered before the writing tests, while the order of presentation of the writing sub-tests was randomized to avoid fatigue biases.

## Results

We computed three MANOVAs to assess whether the two groups (high vs. low performance in the WM updating task) differed in the three subgroups of writing tests: writing speed, orthographic skills, and expressive writing. These MANOVAs were followed up with a discriminant analysis to determine the contribution of the different sub-tests in differentiating the two groups.

### Writing Speed

The 2 (group) × 3 (writing speed sub-tests) MANOVA revealed a main effect of group, *F*(3, 42) = 7.81, *p* < 0.001, and η*_*p*_*^2^ = 0.358. As shown in Table 1, children with high competence in the WM updating task performed significantly better than children with low performance in the WM updating task on each of the three writing speed tests.

**TABLE 1:** Descriptive statistics and results of the univariate tests comparing the two groups on the writing speed tests.

**Task**	**Group**	***M***	***SD***	***F*(1, 44)**	***p***	**η *_*p*_*^2^**
*le*	Low	56.10	14.82	8.53	0.005	0.162
	High	73.04	22.83			
Uno (One)	Low	78.67	20.52	6.14	0.017	0.122
	High	91.96	15.86			
Numbers in words	Low	79.24	15.35	22.39	<0.001	0.337
	High	100.08	14.48			

*Low, Low competence in the WM updating task; High, high competence in the WM updating task.*

### Orthographic Skills

Although the 2 (group) × 4 (orthographic skills sub-tests) MANOVA revealed no main effect of group, *F*(4, 41) = 1.29, *p* = 0.289, and η*_*p*_*^2^ = 0.112, there were significant differences between the two groups in three of the four sub-tests. As shown in Table 2, children with high competence in the WM updating task performed significantly better than children with low performance in the WM updating task in the dictation of words, non-words, and sentences with homophonic and non-homographic words.

**TABLE 2:** Descriptive statistics and results of the univariate tests comparing the two groups on the orthographic tests.

**Task**	**Group**	***M***	***SD***	***F*(1, 44)**	***p***	**η *_*p*_*^2^**
Dictation of a text	Low	10.17	8.39	5.24	0.027	0.106
	High	5.83	4.07			
Dictation of words	Low	16.36	15.42	4.28	0.044	0.089
	High	8.95	8.35			
Dictation of non-words	Low	25.26	12.24	2.31	0.136	0.050
	High	19.90	11.64			
Dictation of sentences	Low	6.27	6.50	4.88	0.032	0.100
	High	3.06	3.02			

*Low, Low competence in the WM updating task; High, high competence in the WM updating task. For all four sub-tests the values reported refer to the percentages of errors.*

### Expressive Writing

The 2 (group) × 4 (expressive skills sub-tests) MANOVA revealed a main effect of group, *F*(4, 41) = 2.80, *p* = 0.038, and η*_*p*_*^2^ = 0.214. As shown in Table 3, children with high competence in the WM updating task committed significantly fewer errors and wrote significantly more words in the description test than children with a low performance in the WM updating task.

**TABLE 3:** Descriptive statistics and results of the univariate tests comparing the two groups on the expressive writing tests.

**Task**	**Group**	***M***	***SD***	***F*(1, 44)**	***p***	**η *_*p*_*^2^**
Narration (*n* of words)	Low	40.20	16.55	1.04	0.313	0.023
	High	45.00	15.31			
Narration (% of errors)	Low	6.56	7.13	3.33	0.075	0.070
	High	3.61	3.48			
Description (*n* of words)	Low	24.84	11.13	5.30	0.026	0.108
	High	32.25	10.64			
Description (% of errors)	Low	6.30	7.47	6.81	0.012	0.134
	High	1.95	3.41			

*Low, Low competence in the WM updating task; High, high competence in the WM updating task.*

### Discriminant Analysis

To determine the contribution of the different sub-tests in differentiating the two groups, we computed a discriminant analysis. The resulting discriminant function explained 42,8% of the variance, canonical *R*^2^ = 0.654, and significantly differentiated the two groups, *χ*^2^(11) = 21.48, *p* = 0.029. The classification results showed that overall, 80.4% of cases were correctly classified in the two groups. Children in the high WM group were classified with higher accuracy (88.0%) than children in the low WM group (71.4%). The structure matrix reported in Table 4 shows the relative importance of each predictor.

**TABLE 4:** Structure matrix of the discriminant analysis.

**Predictors**	**Function 1**
Numbers in words	0.825
*le*	0.510
Description (% of errors)	–0.455
Uno (One)	0.432
Description (*n* of words)	0.402
Dictation of a text (% of errors)	–0.399
Dictation of sentences (% of errors)	–0.385
Dictation of words (% of errors)	–0.361
Narration (% of errors)	–0.318
Dictation of non-words (% of errors)	–0.265
Narration (*n* of words)	0.178

## Discussion

The purpose of this study was to investigate the relationship between WM and writing skills by comparing the performance in different writing tests of children (grades 3–5) with low and high scores on a WM updating test. The results suggest that WM, and especially its active processes, plays a crucial role in a complex learning task such as writing.

Starting from the grapho-motor processes of writing, measured by three writing speed tests with different levels of difficulty (Tressoldi et al., 2013), we observed that children with high performance in the WM updating test also showed faster writing than children with low performance in the WM updating test. The first two tests (repeated writing of “*le*” and “uno”) refer mainly to the fluency of the graphic gesture, which in turn is closely related to the speed with which one can write. In the third test, writing numbers in words is required. In this case, not only a good level of grapho-motor automaticity is required, but also a good ability of the child to access spelling knowledge. Indeed, it is necessary to recall the correct orthographic representation of the “word-numbers” (Tressoldi et al., 2013) and this implies a greater involvement of WM. In this third writing speed test we find, in fact, the highest effect size for the significant difference in performance between the two groups.

In terms of orthographic skills, children with high performance on WM updating performed better on the three more complex orthographic tests (dictation of words, a text, and sentences) than children with low performance on WM updating. These three tests involve not only subvocal repetition but also constant access to orthographic knowledge, which requires greater involvement of active WM. As long as a child is not using automated spelling processes that are not stored in long-term memory, there is a high cost in terms of attentional resources (Graham et al., 1997). Children with low updating performance have a poor ability to coordinate and orient attentional resources, which is reflected in poorer performance (in terms of spelling errors) on writing tests (Graham et al., 1997; Carretti et al., 2009).

In the fourth orthographic subtest – dictation of non-words – no significant difference was found between the two groups. This is probably because this test is mainly based on subvocal repetition of the syllables that make up the non-words, exploiting the writing strategy of letter-by-letter transformation of the phonological path (two-way model of writing, see Coltheart, 1981) and therefore the most passive component of the WM.

Regarding expressive writing, a significant difference was found between the two groups in the percentage of errors in the description test. Such as in dictation, also in the case of spontaneous writing, access to orthographic skills is involved and requires resources from the active WM. Moreover, expressive writing tests ask for various other cognitive processes such as idea generation, planning, and revision, which require continuous control of the WM (Cornoldi et al., 2010). Moreover, in the description test, children with low updating performance did worse in terms of production (number of words written) than children with high updating performance, while no significant differences between the two groups were found for the narration test. We can hypothesize that the narrative task is more dynamic than the description test and it may require less creative effort, making it less complex for children with low WM updating performance.

To sum up, most of the sub-tests contribute in differentiating the two groups, but as shown by the discriminant analysis, the most important one is writing numbers in words. We can hypothesize that this test implicates a major load on the active component of WM because it not only requires coordination between graphomotor skills and spelling knowledge, but also needs a constant updating of the information during its execution. Once the child has written a number, s/he had to replace this information with a new one: the following number.

A limitation of the study is related to the rather small number of participants. However, this is due to the complex health situation we faced. The health emergency related to the spread of COVID-19 led to the closure of schools and the interruption of data collection on 20 February 2020. The project actually envisaged the inclusion of five more classes.

Despite this limitation, the present research has allowed us to better understand the role of the active components of WM in writing performance. In tests of writing speed and in more complex tasks that require not only good phoneme-grapheme conversion skills but also good access to spelling knowledge, the active components of WM are shown to play an important role. Indeed, poor performance in updating WM is indicative of poor ability to coordinate the attentional control resources (Carretti et al., 2009) involved in the different writing processes: graphomotor processes, transcription (phoneme-grapheme conversion and access to spelling knowledge), and higher-order processes involved in written expression.

These results could have important implications both in educational and clinical contexts. In the educational area we suggest to pay attention to the improvement of WM in order to obtain positive effects on writing and even on the graphomotor aspects of writing. In the clinical context, these results suggest that an intervention for dysorthography should not exclude WM training. In other words, if we want to obtain an improvement in a complex learning task such as writing, we have to train and improve also the cognitive processes that are involved, such as active WM.

In future studies, it would be interesting to investigate the relationship between writing performance and WM by using a word span test (passive WM processes) in addition to an updating test (active WM processes). This would allow us to determine which type of WM test is better at capturing differences in performance across the different types of writing processes.

## Data Availability Statement

The raw data supporting the conclusions of this article will be made available by the authors, without undue reservation.

## Ethics Statement

The studies involving human participants were reviewed and approved by Comitato di Bioetica dell’Ateneo (CBA) dell’Università degli Studi di Torino. Written informed consent to participate in this study was provided by the participants’ legal guardian/next of kin.

## Author Contributions

FD carried out the research. SS computed the statistical analysis. CT contributed to the interpretation of the results. FD and SS wrote the manuscript with input from all authors. AR coordinated the research.

## Conflict of Interest

The authors declare that the research was conducted in the absence of any commercial or financial relationships that could be construed as a potential conflict of interest.

## Publisher’s Note

All claims expressed in this article are solely those of the authors and do not necessarily represent those of their affiliated organizations, or those of the publisher, the editors and the reviewers. Any product that may be evaluated in this article, or claim that may be made by its manufacturer, is not guaranteed or endorsed by the publisher.
